# Coffee and Tea Consumption and the Contribution of Their Added Ingredients to Total Energy and Nutrient Intakes in 10 European Countries: Benchmark Data from the Late 1990s

**DOI:** 10.3390/nu10060725

**Published:** 2018-06-05

**Authors:** Edwige Landais, Aurélie Moskal, Amy Mullee, Geneviève Nicolas, Marc J. Gunter, Inge Huybrechts, Kim Overvad, Nina Roswall, Aurélie Affret, Guy Fagherazzi, Yahya Mahamat-Saleh, Verena Katzke, Tilman Kühn, Carlo La Vecchia, Antonia Trichopoulou, Elissavet Valanou, Calogero Saieva, Maria Santucci de Magistris, Sabina Sieri, Tonje Braaten, Guri Skeie, Elisabete Weiderpass, Eva Ardanaz, Maria-Dolores Chirlaque, Jose Ramon Garcia, Paula Jakszyn, Miguel Rodríguez-Barranco, Louise Brunkwall, Ena Huseinovic, Lena Nilsson, Peter Wallström, Bas Bueno-de-Mesquita, Petra H. Peeters, Dagfinn Aune, Tim Key, Marleen Lentjes, Elio Riboli, Nadia Slimani, Heinz Freisling

**Affiliations:** 1 UMR Nutripass, IRD-UM-Sup’Agro, 34394 Montpellier, France; edwige.landais@ird.fr; 2 Nutrition and Metabolism Section, International Agency for Research on Cancer, 69372 Lyon, France; MoskalA@iarc.fr (A.M.); amy.mullee@ucd.ie (A.M.); nicolasg@iarc.fr (G.N.); gunterM@iarc.fr (M.J.G.); huybrechtsi@iarc.fr (I.H.); n.popovic@orange.fr (N.S.); 3 School of Public Health, Physiotherapy and Sports Science, Woodview House, University College Dublin, Belfield, Dublin 4, Ireland; 4 Department of Public Health, Section for Epidemiology, Aarhus University, Bartholins Alle 2, room 2.26, DK-8000 Aarhus, Denmark; ko@ph.au.dk; 5 Danish Cancer Society Research Center, Diet, Genes and Environment, Strandboulevarden 49, DK-2100 Copenhagen, Denmark; roswall@cancer.dk; 6 Inserm CESP U1018, Gustave Roussy, Université Paris-Sud, Paris-Saclay, 94800 Villejuif, France; AURELIE.AFFRET@gustaveroussy.fr (A.A.); Guy.FAGHERAZZI@gustaveroussy.fr (G.F.); Yahya.MAHAMAT-SALEH@gustaveroussy.fr (Y.M.-S.); 7 German Cancer Research Center (DKFZ), Division of Cancer Epidemiology, 69120 Heidelberg, Germany; V.Katzke@Dkfz-Heidelberg.de (V.K.); t.kuehn@Dkfz-Heidelberg.de (T.K.); 8 Hellenic Health Foundation, 115 27 Athens, Greece; carlo.lavecchia@unimi.it (C.L.V.); atrichopoulou@hhf-greece.gr (A.T.); valanou@hhf-greece.gr (E.V.); 9 Department of Clinical Sciences and Community Health, Università degli Studi di Milano, 20122 Milano, Italy; 10 Molecular and Nutritional Epidemiology Unit, ISPO Cancer Prevention and Research Institute, 50139 Florence, Italy; c.saieva@ispo.toscana.it; 11 A.O.U. FEDERICO II, 80131 Naples, Italy; masantuc@unina.it; 12 Epidemiology and Prevention Unit Fondazione IRCCS Istituto Nazionale dei Tumori, 20133 Milan, Italy; Sabina.sieri@istitutotumori.mi.it; 13 Department of Community Medicine UiT, The Arctic University of Norway, 9037 Tromsø, Norway; tonje.braaten@uit.no (T.B.); guri.skeie@uit.no (G.S.); 14 Department of Community Medicine, Faculty of Health Sciences, University of Tromsø, The Arctic University of Norway, 9037 Tromsø, Norway; Elisabete.Weiderpass@kreftregisteret.no; 15 Department of Research, Cancer Registry of Norway, Institute of Population-Based Cancer Research, NO-0304 Oslo, Norway; 16 Department of Medical Epidemiology and Biostatistics, Karolinska Institutet, SE-171 77 Stockholm, Sweden; 17 Genetic Epidemiology Group, Folkhälsan Research Center and Faculty of Medicine, University of Helsinki, 00014 Helsinkiv, Finland; 18 Navarra Public Health Institute, Pamplona, Spain IdiSNA, Navarra Institute for Health Research, 31003 Pamplona, Spain; me.ardanaz.aicua@cfnavarra.es; 19 CIBER Epidemiology and Public Health CIBERESP, 28029 Madrid, Spain; mdolores.chirlaque@carm.es (M.-D.C.); miguel.rodriguez.barranco.easp@juntadeandalucia.es (M.R.-B.); 20 Department of Epidemiology, Regional Health Council, IMIB-Arrixaca, 30008 Murcia, Spain; 21 Department of Health and Social Sciences, Universidad de Murcia, 30008 Murcia, Spain; 22 EPIC Asturias, Public Health Directorate, Asturias, 33006 Oviedo, Spain; joseramon.quirosgarcia@asturias.org; 23 Unit of Nutrition, Environment and Cancer, Catalan Institute of Oncology, 08908 Barcelona, Spain; paujak.ico@gmail.com; 24 Escuela Andaluza de Salud Pública. Instituto de Investigación Biosanitaria ibs, 18011 Granada, Spain; 25 Hospitales Universitarios de Granada, Universidad de Granada, 18014 Granada, Spain; 26 Clinical Science, Lund University, SE-221 00 Lund, Sweden; louise.brunkwall@med.lu.se (L.B.); peter.wallstrom@med.lu.se (P.W.); 27 Department of Internal Medicine and Clinical Nutrition, The Sahlgrenska Academy, University of Gothenburg, SE-405 30 Gothenburg, Sweden; ena.huseinovic@gu.se; 28 Public Health and Clinical Medicine, Nutritional Research, Umeå University, and Arctic Research Centre at Umeå University, SE-901 85 Umeå, Sweden; lena.nilsson@umu.se; 29 Department of Epidemiology and Biostatistics, The School of Public Health, Imperial College London, London W2 1PG, UK; basbuenodemesquita@gmail.com (B.B.-d.-M.); d.aune@imperial.ac.uk (D.A.); e.riboli@imperial.ac.uk (E.R.); 30 Department of Social & Preventive Medicine, Faculty of Medicine, University of Malaya, Kuala Lumpur 50603, Malaysia; 31 University Medical Center Utrecht, 3584 CX Utrecht, The Netherlands; P.H.M.Peeters@umcutrecht.nl; 32 Cancer Epidemiology Unit, Nuffield Department of Population Health, University of Oxford, Oxford OX3 7LF, UK; tim.key@ceu.ox.ac.uk; 33 Strangeways Research Laboratories, Department of Public Health & Primary Care, University of Cambridge, Cambridge CB1 8RN, UK; marleen@srl.cam.ac.uk

**Keywords:** coffee, tea, European Prospective Investigation into Cancer and Nutrition, 24-h dietary recall

## Abstract

Background: Coffee and tea are among the most commonly consumed nonalcoholic beverages worldwide, but methodological differences in assessing intake often hamper comparisons across populations. We aimed to (i) describe coffee and tea intakes and (ii) assess their contribution to intakes of selected nutrients in adults across 10 European countries. Method: Between 1995 and 2000, a standardized 24-h dietary recall was conducted among 36,018 men and women from 27 European Prospective Investigation into Cancer and Nutrition (EPIC) study centres. Adjusted arithmetic means of intakes were estimated in grams (=volume) per day by sex and centre. Means of intake across centres were compared by sociodemographic characteristics and lifestyle factors. Results: In women, the mean daily intake of coffee ranged from 94 g/day (~0.6 cups) in Greece to 781 g/day (~4.4 cups) in Aarhus (Denmark), and tea from 14 g/day (~0.1 cups) in Navarra (Spain) to 788 g/day (~4.3 cups) in the UK general population. Similar geographical patterns for mean daily intakes of both coffee and tea were observed in men. Current smokers as compared with those who reported never smoking tended to drink on average up to 500 g/day more coffee and tea combined, but with substantial variation across centres. Other individuals’ characteristics such as educational attainment or age were less predictive. In all centres, coffee and tea contributed to less than 10% of the energy intake. The greatest contribution to total sugar intakes was observed in Southern European centres (up to ~20%). Conclusion: Coffee and tea intake and their contribution to energy and sugar intake differed greatly among European adults. Variation in consumption was mostly driven by geographical region.

## 1. Introduction

Coffee and tea are the most widely consumed nonalcoholic beverages across the world [1,2]. Both beverages contain various antioxidants and phenolic compounds such as flavonoids or caffeine, some of which have been shown to have anticancer properties in laboratory conditions [3,4,5,6].

According to the third expert report of the World Cancer Research Fund (WCRF) and the Continuous Update Project (CUP), the evidence on the associations between cancer and the intakes of tea, and for many cancer sites, of coffee, were too limited in amount, consistency, and/or quality to draw conclusions, except for a probable decreased risk for cancers of the liver and endometrium among regular coffee drinkers [3,7].

Several systematic reviews and meta-analyses conducted subsequently also reported inconsistent results for the potential association of coffee or tea on certain types of cancers such as prostate, lung, colorectal, oesophageal, renal, or breast cancers. Indeed, whilst some of the studies reported inverse associations for tea or coffee (e.g., coffee and liver or prostate cancers, tea and lung cancer) [8,9,10,11,12,13], others did not observe any significant adverse or potential protective effects of such beverages [14,15,16,17,18,19].

A monograph conducted by the International Agency for Research on Cancer (IARC) in 2016 evaluating the carcinogenicity of drinking coffee to humans concluded that it was unclassifiable as to its carcinogenicity to humans [20].

Differences in tea- and coffee-drinking habits (e.g., green tea, black tea, with caffeine, decaffeinated) as well as the preparation processes, amount consumed, and additions such as sugar/milk are likely to vary by population and countries and could contribute to the inconsistencies found between studies comparing tea and coffee consumption and the risk of chronic diseases. Furthermore, the use of different assessment methods, such as distinct food frequency questionnaires, different variable definitions (e.g., food classification, serving sizes), or levels of detail to describe foods, may impede comparisons between studies [21].

Our main objective was to describe coffee and tea intake in men and women across 27 centres in the European Prospective Investigation into Cancer and Nutrition (EPIC) study using standardized 24-h dietary recall (24-HDR) data. We also estimated variation in intake levels according to selected sociodemographic, lifestyle, and anthropometric characteristics of study participants, and assessed the relative contribution of coffee and tea to intakes of total energy and selected nutrients (total sugars, calcium, magnesium, phosphorus).

## 2. Materials and Methods

### 2.1. Setting and Subjects

EPIC is a multicentre prospective cohort study investigating the association between diet and cancer and other chronic diseases in 23 centres in ten countries: Denmark, France, Germany, Greece, Italy, the Netherlands, Norway, Spain, Sweden, and the UK [22,23]. EPIC participants were mostly recruited from the general population between 1992 and 1998 and included 521,330 men and women aged 35–70 years; exceptions were France (health insurance members), Utrecht (The Netherlands) and Florence (Italy) (participants of breast cancer screening), and some centres in Spain and Italy (mostly blood donors). In the UK, a cohort consisting predominantly of vegetarians (‘health-conscious’ in Oxford) was considered separately from a ‘general population’ group recruited by general practitioners in Cambridge and Oxford. Most centres recruited both men and women, except Norway, France, Utrecht, and Naples, where only women were recruited. Details of the methods of recruitment and study design have been published previously [22,24,25]. All participants provided written informed consent, and the project was approved by ethical review boards of the IARC and local participating centres. In the present study, the initial 23 EPIC centres were redefined into 27 regions according to a geographical south–north gradient and relevant to analyses of dietary consumption and patterns [23].

The calibration substudy nested within the EPIC cohort was undertaken between 1995 and 2000 with the aim to partially correct for attenuation in diet–disease associations due to measurement errors. This has been obtained by rescaling the country-specific individual dietary intakes against the same reference dietary measurement obtained using a highly standardized 24-h dietary recall (24-HDR) [26]. The calibration population sample consisted of 36,994 participants, representing a random sample (~8%) of the total EPIC cohort, stratified by age, sex, and centre. Details of the population characteristics of the calibration study have been published previously [23,27,28,29]. In brief, each participant completed a single 24-HDR during a face-to-face interview, except in Norway, where it was conducted through a validated phone interview alternative [30]. A computer-based interview programme, named EPIC-Soft (recently renamed GloboDiet; IARC, Lyon, France), was developed to conduct standardized 24-HDR interviews [31,32] with the same structure and interview procedure across countries. The interviews were conducted over different seasons and days of the week. For logistical constraint reasons, interviews recalling diet on Saturday were conducted on Monday (instead of Sunday) in most countries, whereas for all other days of the week, the interviews were conducted the following day. Time and place of consumption were also collected.

### 2.2. Dietary Variables

The common food group classification used in the EPIC-Soft software, which has been described previously [23], was used to divide the overall coffee and tea group into four different subgroups as follows: coffee, split into three subgroups regarding caffeine content (with caffeine, partially decaffeinated, decaffeinated); tea, either black or green; herbal tea; and chicory and substitutes. Anything added to these beverages, e.g., milk or milk substitutes, sugar, and honey, was also taken into consideration, in order to evaluate the overall contribution of coffee and tea with their added ingredients to total energy and selected nutrients’ intake (alcohol was a negligible ingredient to coffee in all cohorts). The beverages are expressed in grams per day as complete beverages (i.e., including the water for diluted beverages or reconstituted beverages from powder). The overall coffee and tea intake of individuals on the recall day was calculated by summing the amount of these four groups.

Places where coffee and tea could potentially be consumed were recorded as home, work, fast-food restaurant, bar, cafeteria, restaurant, friends’ home, school, street, car/boat/plane, and other. These options were common across centres. After considering their distribution, some of these categories were merged as follows: work, school, and cafeteria into ‘work’; other, street, and car/boat/ plane into ‘other’; and fast-food restaurant with restaurant. The resulting places of consumption were: home, work, bar, restaurant, friends’ place, and other place.

### 2.3. Nutrient Databases

Energy and nutrient intakes were estimated by means of standardized nutrient databases developed through the EPIC Nutrient DataBase (ENDB) project. Only relevant nutrients (sugar, calcium, magnesium, phosphorus) with regards to coffee and tea and their related added ingredients are reported. The rationale and procedures used to improve between-country comparability of the 26 nutrients included in this database are described elsewhere [33].

### 2.4. Nondietary Variables

Data on other lifestyle factors, including education (none or primary, secondary/technical, and university degree; completeness >98%), total physical activity (inactive, moderately inactive, moderately active, and active; completeness >86%) [34], and smoking status (never, former, current; completeness >98%), were collected at baseline through standardized questionnaires and clinical examinations and have been described elsewhere [22,23,35]. In most centres, age as well as body weight and height were self-reported by the participants during the 24-HDR interview. Individuals were classified according to age categories (35–44, 45–54, 55–64, 65–74 years) and body mass index (BMI; based on self-reported data) categories (BMI < 25 kg/m^2^, BMI 25 to <30 kg/m^2^, BMI ≥ 30 kg/m^2^; no missing data). The time interval between the baseline questionnaires and the 24-HDR interview varied by country, ranging from one day to three years [23].

### 2.5. Statistical Methods

Centre-specific arithmetic means of coffee and tea intakes and standard errors of the mean (SEM) were calculated using generalized linear models, stratified by EPIC centre and sex. Fully adjusted models were adjusted for age, total energy intake, height, and weight (except for analyses stratified on BMI) and were weighted by season and day of recall to control for different distributions of 24-HDR interviews across seasons and days of the week. Means were also calculated for each type of coffee and tea as well as for decaffeinated versus caffeinated (including partially decaffeinated) coffee. If fewer than 20 persons were represented in a cross-classification (for example, centre, sex, and age group), the least-square mean was not reported in the table.

In order to compare means of coffee and tea across centres by categories of age, education, BMI, physical activity, and smoking status, we fitted regression models that included an interaction term between centre and each of the potentially associated factors at a time, to test whether the association of coffee and tea consumption with these factors differed across centres. These analyses were adjusted for age, total energy intake, height, and weight and weighted by season and day of recall, separately for men and women. Participants with missing data were omitted. Type III statistics of the GENMOD procedure in SAS were used to examine the partial effect of each variable; that is, the significance of a variable with all the other variables in the model. Tests for trends were computed across categories by using a score variable (from 1 up to the number of categories of a given variable).

The relative contribution of coffee and tea intake (overall and by type) to total energy and selected nutrient intakes (sugar, calcium, magnesium, phosphorus) were calculated by centre as the mean percentage of intake, stratified by centre; adjusted for sex, height, and weight; and weighted by season and weekday.

All the analyses were performed using SAS (version 9.4, SAS Institute, Cary, NC, USA).

## 3. Results

A total of 36,018 subjects with 24-HDR data were included in this analysis, after exclusion of 958 subjects aged under 35 or over 74 years because of low participation in these age categories and of 18 subjects without lifestyle variable data.

### 3.1. Coffee and Tea Intakes

The adjusted mean daily intake of coffee and tea varied widely across centres, ranging from 174 g/day and 170 g/day for men and women, respectively, in Greece to 1468 g/day and 1321 g/day in the UK general population (Table 1 for men and Table 2 for women). Overall, Northern European countries tended to drink more coffee and tea compared to Southern European countries (see Supplemental Materials, Table S1).

When describing consumption for the four different coffee and tea groups, the adjusted mean daily intake of coffee ranged from 107 g/day in Greek men (which corresponded to 0.9 cups) to 1016 g/day for men living in Aarhus (Denmark) (which corresponded to 5.5 cups) (Table 1) and from 94 g/day for Greek women (which corresponded to 0.6 cups) to 781 g/day for women from Aarhus (Denmark) (which corresponded to 4.4 cups) (Table 2). Among men, tea intake ranged from 18 g/day in San Sebastian (Spain) (which corresponded to 0.1 cups) to 928 g/day in the UK general population (which corresponded to 4.9 cups), and among women from 14 g/day in Navarra (Spain) (which corresponded to 0.1 cups) to 788 g/day in the UK general population (which corresponded to 4.3 cups). Across centres, the lowest consumption of herbal tea was observed in Umeå (Sweden) (0 g/day and 7 g/day for men and women, respectively) and the highest one in Germany (128 g/day for men in Potsdam and 202 g/day for women in Heidelberg). For both men and women, the lowest consumption of chicory and substitutes was reported in Sweden and Denmark, and the highest in UK health-conscious individuals (Table 1 and Table 2).

Overall, in all centres but those in the UK, the amount of coffee consumed was higher than the amount of tea for both sexes.

### 3.2. Proportion of Consumers

In comparison with all centres, Greece had the highest proportion of individuals not consuming coffee nor tea over the previous day (27% and 31% for men and women, respectively), and Aarhus (Denmark) for men and Utrecht (The Netherlands) for women had the lowest proportion of nonconsumers (0.9% and 0.4%, respectively) (see Supplemental Materials, Figures S1 and S2). The proportion of men drinking only tea the previous day was the lowest in Ragusa (Italy) (0.6%) and the highest in the UK general population (23%). Women from Naples (Italy) and Navarra (Spain) had the lowest proportion of tea-only drinkers the previous day (0.7% in both cases) and the UK health-conscious population had the highest proportion (30%). The proportion of men and women drinking coffee only over the previous day was the lowest in the UK general population (10% and 12%, respectively) and the highest for both Italian men and women (Ragusa 87% and Naples 86%, respectively). Apart from in the UK, most of the men were coffee drinkers only. The same pattern was found for women in the UK as well as in The Netherlands.

Among coffee consumers from both sexes, the large majority of coffee consumed was coffee with caffeine (see Supplemental Materials, Figures S3 and S4). Overall, the mean percentage of decaffeinated coffee consumers slightly differed between sexes, with women tending to drink more decaffeinated coffee than men (8.8% vs. 6.0%). No south–north gradient was observed for the consumption of decaffeinated coffee. In Granada (Spain), men and women were the highest consumers of decaffeinated coffee (33% and 38%, respectively). In Malmö (Sweden), both men and women were the lowest consumers of decaffeinated coffee (0.3% and 0.6%, respectively).

### 3.3. Place of Consumption

**Figure 1:**
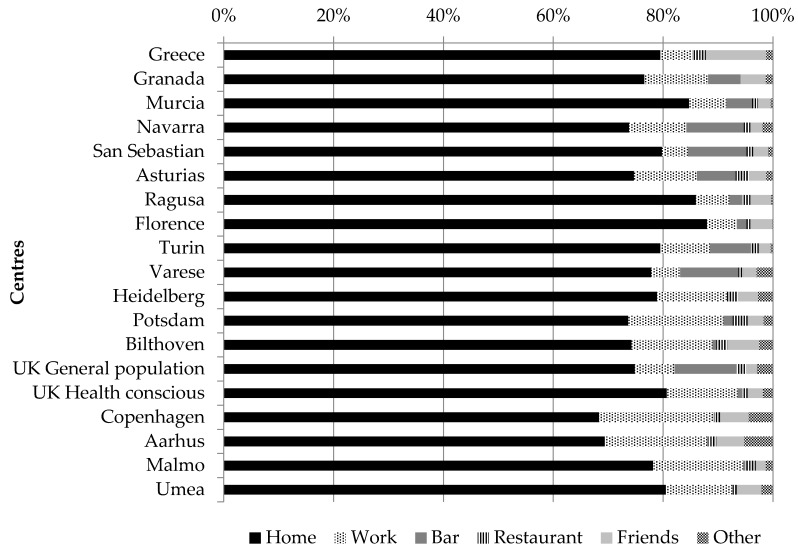
Proportion of coffee and tea consumption at different places of consumption, among men across EPIC centres; fully adjusted models among consumers only; “friends” refers to friends’ place.

**Figure 2:**
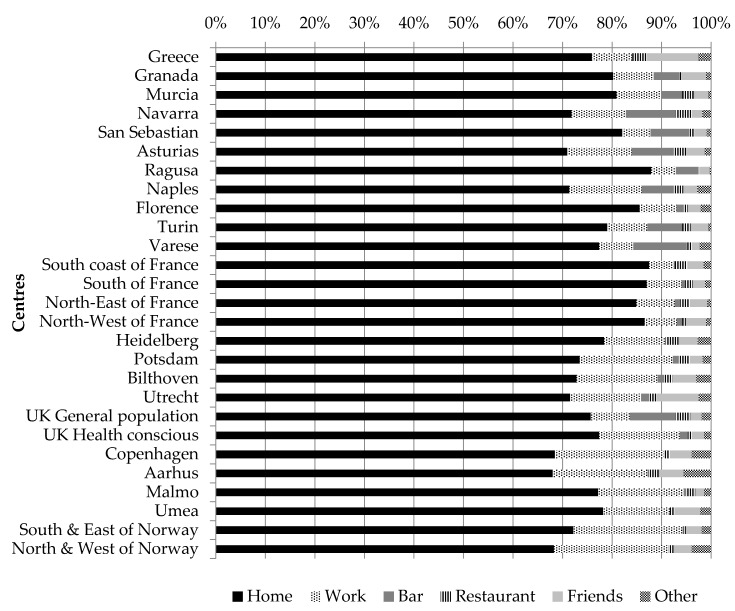
Proportion of coffee and tea consumption at different places of consumption, among women across EPIC centres; fully adjusted models among consumers only; “friends” refers to friends’ place.

When investigating the place of consumption, the large majority of coffee or tea consumed was consumed at home by both women and men. The percentage ranged from over 60% for both sexes in Denmark to almost 90% of all coffee and tea consumed in Italy (for men, the percentage ranged from 68% in Copenhagen (Denmark) to 88% in Florence (Italy), and for women, from 68% in Aarhus (Denmark) to 88% in Ragusa (Italy) (Figure 1 and Figure 2).

The second most important place of consumption was work, for which there was a south–north gradient as overall, for individuals living in the Northern part of Europe, coffee and tea were more frequently drunk at work compared to what was reported in the Southern part. The other important places of consumption were “bar” and “friends’ place”, for which a south–north gradient was observed. Indeed, for women living in the Northern part of Europe, coffee and tea were more frequently consumed at a friends’ place rather that at a bar. The opposite pattern was observed for women living in South Europe, except for Greek women and women living in the South of France. A similar pattern was observed among men.

### 3.4. Sociodemographic Factors

When studying the age trends, overall, coffee and tea intake was significantly associated with age (*p* < 0.0001 in both sexes). Stratified by centre, a linear trend between coffee and tea consumption and age was only significant among four out of the 23 centres (Table 3), which could be related to lack of power due to stratification. In Greece and Florence (Italy), older men tended to drink significantly more coffee and tea compared to the younger ones. On the contrary, younger men from Malmö (Sweden), as well as younger women from Navarra (Spain), drank significantly more coffee and tea than their older counterparts on the day of the recall.

Education across all centres was significantly associated with coffee and tea consumption among both men and women (*p* < 0.005 and *p* < 0.0001, respectively). Overall, the amount of coffee and tea consumed was higher with higher education. Yet, when stratified by centre, the linear trend between coffee and tea intake and education was significant only in men from the UK general population (the less educated tended to drink more coffee and tea), as opposed to women from the South of France, Copenhagen (Denmark), and Umeå and Malmö (Sweden), where the more educated tended to drink more coffee and tea on the day of the recall compared to the less educated women (Table 4).

### 3.5. Lifestyle Factors

Lifestyle factors such as smoking (*p* < 0.001 for men and women) and physical activity (*p* < 0.01 for men and *p* = 0.03 for women) were both associated with coffee and tea consumption. These two factors were still significant when considering coffee and tea separately in both men and women. Whilst there was a clear pattern for smoking, where current smokers drank more coffee and tea compared to “never” smokers, a similarly consistent pattern was not found for physical activity (see Supplemental Materials, Tables S2 and S3). Nevertheless, significant linear trends were found among men in Murcia (Spain, *p* = 0.02), Bilthoven (The Netherlands, *p* = 0.04), and Copenhagen (Denmark, *p* = 0.04), where active men tended to drink ~100 g/day less coffee and tea combined compared to inactive men. The opposite was observed for men from the UK general population (*p* < 0.05). Similar patterns were observed in women in these very same centres, although respective linear trends were statistically nonsignificant (all *p* > 0.13).

The overall association between BMI and coffee and tea consumption was not significant among women (*p* = 0.06), but was significant among men (*p* < 0.001), although with no clear pattern except for men from Potsdam (Germany), where normal-weight men tended to drink significantly more coffee and tea compared to obese men (Table S4).

### 3.6. Contribution to Energy and Micronutrients

The contribution of coffee and tea along with their added ingredients (i.e., milk, sugar, honey, etc.) to energy, sugar, calcium, magnesium, and phosphorus intakes was the lowest in Norway. The contribution of coffee and tea to energy intake ranged from 1.2% in the south and east of Norway to 8.2% in Asturias (Spain) (Table 5). The contribution to sugar intake ranged from 2.5% in the north and west of Norway to 23% in Varese (Italy). Coffee and tea contributed to more than one-fifth of sugar intake in five centres, all of them belonging to the southern centres (Granada, Navarra, Asturias, Naples, and Varese). The contribution of coffee and tea to calcium intake ranged from 3.3% in the north and west of Norway to 33% in Asturias (Spain). As for sugar, in Spain and in most Italian centres, coffee and tea contributed to more than one-fifth of calcium intake, reaching one-fourth and even one-third in some centres. The contribution of coffee and tea to magnesium intake ranged from 8.7% in Greece to 35% in France. Compared to other countries, in France, this contribution was higher and around 30%. The contribution of coffee and tea to phosphorus intake ranged from 1.6% in Norway to 19% in Murcia (Spain).

## 4. Discussion

This is one of the largest population-based studies comparing coffee and tea consumption using a common, detailed, and standardized 24-h dietary recall method across 10 European countries participating in the EPIC study.

The amount of coffee and tea consumed varied widely across countries/centres and according to the type of beverages consumed. Average tea consumption was highest in the UK and lowest in Greece and Spain, while coffee consumption was highest in Denmark and lowest in Greece.

Apart from Greece, the majority of coffee and tea intakes from the previous day was consumed at home. Most coffee drinkers consumed caffeinated coffee.

These results are consistent with studies that used long-term dietary assessment methods in the EPIC cohort [36,37,38]. For coffee, the observed geographical differences might be due to different consumption habits. For instance, in countries such as Denmark, people tend to drink more diluted coffee in larger amounts, whilst in other countries such as Greece or Italy, people tend to drink stronger coffee in smaller amounts (e.g., Turkish coffee or ristretto coffee). Indeed, in Italy, the mean cup of coffee weighed 55 g, whereas in Denmark, the mean cup of coffee weighed 182 g.

Coffee and tea consumption also varied to some extent by sex, age, and education, with the direction of the associations being different across centres. For example, coffee and tea consumption combined decreased with level of education in the UK general population by about 200–300 g/day, comparing the population subgroup with primary education to that with a university degree (Table 4); whereas an opposite trend was observed in the two centers in Sweden (Malmö, Umea) and in Copenhagen (Denmark). In the remaining countries/centers, differences across level of education were less pronounced, which suggests that coffee and tea consumption is driven by country-specific dietary habits rather than characteristics at the individual level. Other studies that have investigated relationships between sociodemographic factors and coffee consumption also reported mixed results. For instance, the National Health and Nutrition Examination Survey (NHANES) 2003–2012 in the US observed that the mean usual intakes of coffee were higher in men than in women, in older versus younger individuals, and in lower- versus higher-educated individuals [39]. Different results were reported from the Japan Collaborative Cohort Study for Evaluation of Cancer Risk (JACC study), in which both men and women with high coffee consumption were younger and better educated [40]. A cross-sectional population-based survey conducted in Poland reported that higher coffee consumers were more likely to be women, younger, and with a medium–higher education [41]. The same study also reported that higher tea consumers were more likely to be women. These mixed results emphasize the fact that coffee and tea consumption differs with the population under investigation and explain why no homogeneity was found across the different EPIC centres.

In the present study, current smokers compared to former/“never” smokers tended to drink more coffee and tea. Other studies conducted in the US, but focusing on coffee only, reported that lifestyle factors such as smoking were related to coffee consumption. Also, in the National Institute of Health-American Association of Retired Persons Diet and Health Study, coffee drinkers where more likely to smoke [2]. A more recent study, also conducted in the US but using the NHANES 2003–2012 data, reported that the mean intake of coffee was higher among smokers versus “never” smokers [39]. The same pattern was also observed in Japan [40], Singapore [42], and Brazil [43].

Overall, BMI was associated to coffee and tea consumption among men, but with no clear patterns, and was not associated with coffee and tea consumption among women. This result, albeit different from what is generally reported in the literature [37,41,44,45], was not unexpected, considering the cross-sectional design and the use of a single 24-h dietary recall, and that the development of overweightness or obesity is a life course process.

The contribution of coffee and tea to sugar and calcium intakes was higher in Italy and Spain compared to other countries, reflecting different consumption habits and suggesting that in Southern European countries, people tended to add (more) sugar and milk, which both contribute to total sugar intake, to their coffee and tea. In those two countries, coffee and tea, with their added ingredients, contributed to about 20% to the overall sugar intakes, whilst in Norway, coffee and tea contributed to less than 3%. Given these results, it is recommended to consider both coffee and tea as potential major sources of sugar intake (free/added sugars) in dietary monitoring and public health surveillance. There are health concerns regarding added/free sugar consumption, and compared to carbonated soft drinks, coffee and tea with their added ingredients receive less attention. In a more positive sense, this also applies to coffee and tea as a source of calcium, where the milk added is rarely considered as a source of calcium.

The present study was based on a single 24-HDR and therefore did not reflect usual intakes of individuals. Hence, the interpretation of nonconsumers should be performed with caution due to the day-to-day variability. Indeed, the prevalence of tea or coffee nonconsumers was higher compared to the same prevalence measured with the EPIC country-specific Food Frequency Questionnaire assessing food intakes over the past 12 months [46]. However, considering the large sample size, except in Ragusa, and the fact that individuals usually drink such beverages on a daily basis, the population mean consumption levels should have been reasonably well captured. Indeed, when comparing the results of the present calibration study to the EPIC long-term consumption data, similar patterns were found [36,37,38]. Moreover, the standard error of the mean should be interpreted with caution because it is most likely overestimated due the day-to-day variation in consumption levels.

Data for the current study were collected in the mid to late 1990s, and coffee and tea intakes may have changed over time. Compared to more recent surveys conducted between 2003 and 2011 in Germany, Denmark, Spain, the UK, Italy, The Netherlands, and Sweden, where a similar dietary assessment method was used, i.e., 24-h dietary recalls, coffee intake in our study was lower, whilst tea intake was higher [47]. Such comparisons indicate that our study may serve as a common benchmark to evaluate trends in coffee and tea consumption over time in these countries.

However, some caution is warranted because the EPIC study populations were not necessarily representative of the corresponding national populations, and in several countries, they tended to be more “health-conscious”.

Although the information about coffee was detailed, as individuals were asked to specify whether coffee was with caffeine or decaffeinated, the EPIC Nutrient DataBase does not contain information on caffeine content. Hence, for instance, one cup of coffee in Italy—where a 60-mL cup of espresso contains approximatively 80 mg of caffeine [48]—cannot be strictly compared with one cup of coffee in Denmark, where a 200-mL cup of filter coffee contains approximatively 90 mg of caffeine [48]. However, caffeine intake across Europe, as reported in the European Food Safety Authority’s fact sheets on caffeine [48], roughly confirm our findings based on consumed quantity of the beverages. For example, the estimated caffeine intakes in Greece (~30 mg/day) and Spain (67 mg/day) were lower as compared to Denmark (~320 mg/day) or Germany (~238 mg/day) [48]. The same reasoning applies for tea, as the different types of tea (green, white, black) differ in caffeine content [49]. The assessment of caffeine intake is of importance and therefore there is a need for collecting more detailed data, to add caffeine content in food composition tables or to use biomarkers, such as the dimethylxanthines theophylline or paraxanthine, in order to enable a more objective assessment of caffeine intake [49]. Additionally, the brewing method might also be considered when collecting data because of the consequences on the content of diterpenes [50] that have an anticarcinogenic activity [6].

The health benefits of coffee and tea consumption are still controversial [15,17,19]. Therefore, the use of a standardized method such as the one used in the present study, but with repeated assessments, to collect comparable dietary data across countries is of interest as it might help to investigate better associations between coffee and tea consumption and health outcomes. Moreover, such a method provides data that is not only geographically comparable, but is also comparable over time.

## 5. Conclusions

Levels of coffee and tea intake, and their contribution to energy and sugar intake, differed greatly among European adults. Variation in consumption was mostly driven by geographical region and to a lesser extent by individuals’ characteristics.

## Figures and Tables

**Table 1:** Mean daily intake of coffee and tea (g/day) by type in the EPIC calibration study population based on 24-H Dietary Recall among men across EPIC centres ordered from south to north.

Country and Centre		Total Coffee and Tea *		Coffee		Tea *		Herbal Tea		Chicory and Substitutes	
	*n*	Fully Adjusted Mean ^1^	SEM ^2^	Fully Adjusted Mean ^1^	SEM ^2^	Fully Adjusted Mean ^1^	SEM ^2^	Fully Adjusted Mean ^1^	SEM ^2^	Fully Adjusted Mean ^1^	SEM ^2^
Greece	1324	173.5	13.3	106.7	12.2	47.9	9.2	18.3	4.4	0.6	1.8
Spain											
Granada	214	387.3	31.9	316.1	29.4	27.3	22.1	31.7	10.6	12.2	4.3
Murcia	243	302.0	30.0	202.9	27.7	25.1	20.8	53.5	10.0	20.5	4.0
Navarra	444	309.2	22.3	267.2	20.6	18.7	15.4	14.4	7.4	9.0	3.0
San Sebastian	490	270.2	21.4	192.9	19.7	17.7	14.8	28.1	7.1	31.6	2.9
Asturias	386	379.5	23.8	295.0	22.0	23.0	16.5	29.1	7.9	32.4	3.2
Italy											
Ragusa	168	222.6	36.0	160.3	33.2	47.6	25.0	4.9	12.0	9.7	4.8
Florence	271	270.1	28.2	187.2	26.0	45.5	19.6	9.0	9.4	28.4	3.8
Turin	676	260.9	18.0	171.7	16.6	56.3	12.5	13.6	6.0	19.3	2.4
Varese	327	392.6	25.8	277.9	23.8	70.1	17.9	14.6	8.6	29.9	3.5
Germany											
Heidelberg	1034	897.1	14.6	597.7	13.4	164.6	10.1	125.6	4.9	9.3	2.0
Potsdam	1233	843.9	13.2	578.7	12.2	126.8	9.2	128.2	4.4	10.3	1.8
The Netherlands											
Bilthoven	1020	960.5	15.1	698.1	13.9	235.0	10.5	21.6	5.0	5.8	2.0
United Kingdom											
General population	405	1467.7	23.1	523.9	21.3	927.8	16.0	9.5	7.7	6.4	3.1
Health-conscious	113	1222.4	43.9	439.0	40.5	620.5	30.4	113.1	14.6	49.9	5.9
Denmark											
Copenhagen	1356	1152.0	12.7	896.7	11.8	229.9	8.8	25.7	4.2	0.0	
Aarhus	567	1220.8	19.6	1015.5	18.0	184.9	13.6	18.0	6.5	2.3	2.6
Sweden											
Malmö	1421	855.7	13.2	727.1	12.1	133.5	9.1	0.0		0.0	
Umeå	1342	785.6	12.8	626.1	11.8	160.4	8.9	0.0		0.0	

* Either green or black tea, herbal tea excluded. ^1^ Adjusted for age, total energy intake, weight, and height and weighted by season and day of recall. ^2^ SEM: standard error of the mean.

**Table 2:** Mean daily intake of coffee and tea (g/day) by type in the EPIC calibration study population based on 24-H Dietary Recall among women across EPIC centres ordered from South to North.

Country and Centre		Total Coffee and Tea *		Coffee		Tea *		Herbal Tea		Chicory and Substitutes	
	*n*	Fully Adjusted Mean ^1^	SEM ^2^	Fully Adjusted Mean ^1^	SEM ^2^	Fully Adjusted Mean ^2^	SEM ^2^	Fully Adjusted Mean ^2^	SEM ^2^	Fully Adjusted Mean ^1^	SEM ^2^
Greece	1368	170.3	12.5	93.8	10.3	54.5	9.8	20.0	5.5	2.0	3.4
Spain											
Granada	300	425.9	25.8	299.8	21.3	24.4	20.2	78.4	11.4	23.3	7.1
Murcia	304	389.9	25.6	289.3	21.1	20.9	20.1	62.0	11.4	17.7	7.1
Navarra	271	491.2	27.0	433.4	22.3	14.4	21.2	24.5	12.0	18.9	7.4
San Sebastian	244	468.3	28.4	360.8	23.5	20.8	22.3	39.9	12.6	46.8	7.8
Asturias	324	532.8	24.7	454.9	20.4	18.3	19.4	37.6	11.0	22.0	6.8
Italy											
Ragusa	137	201.0	38.1	147.8	31.4	32.4	29.9	12.0	16.9	8.9	10.5
Naples	403	297.2	22.3	226.6	18.4	41.5	17.5	10.3	9.9	18.8	6.1
Florence	783	328.1	15.9	226.3	13.1	54.8	12.5	17.2	7.1	29.7	4.4
Turin	392	312.3	22.4	194.9	18.5	73.3	17.6	21.3	10.0	22.8	6.2
Varese	795	404.1	15.9	262.8	13.1	97.0	12.5	25.1	7.0	19.1	4.4
France											
South coast	620	567.0	17.9	282.8	14.8	147.1	14.1	63.7	8.0	73.4	4.9
South	1425	651.7	11.9	280.7	9.8	228.7	9.3	64.4	5.3	78.0	3.3
Northeast	2059	656.0	9.9	323.3	8.2	200.3	7.8	62.1	4.4	70.3	2.7
Northwest	631	722.9	17.8	365.3	14.7	245.2	13.9	50.4	7.9	62.1	4.9
Germany											
Heidelberg	1087	968.7	13.6	557.6	11.2	193.1	10.7	202.2	6.1	15.8	3.8
Potsdam	1060	815.8	13.7	510.3	11.3	113.2	10.8	178.7	6.1	13.6	3.8
The Netherlands											
Bilthoven	1076	949.0	13.8	591.0	11.4	303.3	10.8	42.4	6.1	12.3	3.8
Utrecht	1870	1050.1	10.4	570.1	8.6	431.9	8.2	39.7	4.6	8.4	2.9
United Kingdom											
General population	570	1321.3	18.6	491.2	15.3	788.4	14.6	34.3	8.2	7.4	5.1
Health-conscious	196	1139.0	31.7	328.1	26.1	601.4	24.9	116.1	14.1	93.4	8.7
Denmark											
Copenhagen	1484	1009.3	11.6	631.7	9.6	315.8	9.1	61.4	5.2	0.5	3.2
Aarhus	510	1109.5	19.7	781.0	16.2	230.2	15.4	95.0	8.7	3.3	5.4
Sweden											
Malmö	1711	805.7	11.0	646.3	9.1	147.6	8.7	11.9	4.9	0.0	
Umeå	1567	704.0	11.2	527.8	9.3	168.9	8.8	7.1	5.0	0.2	3.1
Norway											
South and East	1004	892.8	14.4	643.7	11.8	190.1	11.3	57.1	6.4	1.9	4.0
North and West	793	894.5	16.0	690.9	13.2	135.1	12.6	67.4	7.1	1.1	4.4

* Either green or black tea, herbal tea excluded. ^1^ Adjusted for age, total energy intake, weight, and height and weighted by season and day of recall. ^2^ SEM: standard error of the mean.

**Table 3:** Fully adjusted mean ^1^ daily intake of coffee and tea (g/day) by age group and sex in the EPIC calibration study population based on 24-H Dietary Recall across EPIC centres ordered from south to north.

Country and Centre	Men												Women											
		All		35–44 Years		45–54 Years		55–64 Years		65–74 Years				All		35–44 Years		45–54 Years		55–64 Years		65–74 Years		
	*n*	Mean ^1^	SEM ^2^	Mean ^1^	SEM ^2^	Mean ^1^	SEM ^2^	Mean ^1^	SEM ^2^	Mean ^1^	SEM ^2^	*p*-Trend	*n*	Mean ^1^	SEM ^2^	Mean ^1^	SEM ^2^	Mean ^1^	SEM ^2^	Mean ^1^	SEM ^2^	Mean ^1^	SEM ^2^	*p*-Trend
Greece	1324	173.5	13.3	116.4	37.9	137.9	26.9	184.5	23.1	191.6	22.0	0.034	1368	170.3	12.5	146.2	31.8	169.4	21.3	179.7	21.4	166.3	26.3	0.349
Spain																								
Granada	214	387.3	31.9	377.6	133.3	444.5	64.6	381.3	43.2	317.2	77.6	0.393	300	425.9	25.8	426.8	63.8	475.9	42.7	409.9	40.5	349.4	88.0	0.263
Murcia	243	302.0	30.0	319.8	85.3	342.1	54.6	277.5	41.4	343.7	113.6	0.970	304	389.9	25.6	408.0	49.7	463.4	43.3	351.2	42.0	268.1	144.8	0.177
Navarra	444	309.2	22.3	254.4	86.0	305.6	37.3	322.1	31.4	314.5	71.8	0.169	271	491.2	27.0	603.1	66.8	493.0	44.3	469.2	40.7	385.8	143.4	0.026
San Sebastian	490	270.2	21.4	304.0	46.5	282.9	29.4	264.3	41.0	298.8	119.3	0.754	244	468.3	28.4	421.4	59.5	482.5	45.2	513.7	47.5	454.6	164.6	0.571
Asturias	386	379.5	23.8	453.1	79.5	360.1	40.4	377.3	34.8	414.1	70.1	0.689	324	532.8	24.7	570.6	58.6	593.1	39.5	494.8	39.7	390.7	104.0	0.098
Italy																								
Ragusa	168	222.6	36.0	184.5	103.6	234.9	53.2	240.6	55.7	250.0	246.7	0.110	137	201.0	38.1	197.2	62.6	234.9	70.5	233.0	68.5	171.6	179.0	0.667
Naples													403	297.2	22.3	264.9	68.7	313.2	33.2	303.2	36.6	272.0	73.3	0.938
Florence	271	270.1	28.2	200.1	84.8	246.5	46.0	310.5	41.1	324.3	121.7	0.025	783	328.1	15.9	306.5	49.8	328.2	27.3	333.7	22.1	314.3	68.8	0.702
Turin	676	260.9	18.0	260.6	54.3	268.4	29.7	268.7	25.4	225.6	86.7	0.341	392	312.3	22.4	292.7	66.6	336.4	36.4	302.0	32.1	373.8	134.9	0.269
Varese	327	392.6	25.8	368.0	141.7	364.8	58.1	397.1	30.2	393.4	109.9	0.164	795	404.1	15.9	376.0	48.0	405.4	26.2	414.6	23.9	391.6	49.7	0.571
France																								
South coast													620	567.0	17.9			608.2	28.6	541.4	27.6	497.9	39.8	0.077
South													1425	651.7	11.9	349.5	376.3	662.4	17.9	658.8	18.7	577.0	28.2	0.404
Northeast													2059	656.0	9.9			663.0	15.0	648.4	15.4	619.2	23.8	0.121
Northwest													631	722.9	17.8			762.2	27.5	685.9	27.0	682.5	43.7	0.309
Germany																								
Heidelberg	1034	897.1	14.6	946.8	37.4	949.2	22.9	846.3	21.4	1496.3	241.5	0.324	1087	968.7	13.6	1005.3	22.9	999.2	24.1	951.6	22.8			0.267
Potsdam	1233	843.9	13.2	862.2	36.4	854.8	26.4	834.9	17.3	918.3	64.5	0.464	1060	815.8	13.7	816.5	26.5	879.4	26.6	806.9	19.8	538.2	154.5	0.227
The Netherlands																								
Bilthoven	1020	960.5	15.1	987.7	27.8	989.8	22.9	946.9	25.5	850.8	333.6	0.100	1076	949.0	13.8	926.1	23.9	1030.7	21.1	923.8	26.2	756.2	249.5	0.299
Utrecht													1870	1050.1	10.4			1040.7	17.2	1047.5	15.7	1024.7	22.0	0.522
United Kingdom																								
General population	405	1467.7	23.1	1311.7	73.8	1595.7	40.6	1418.3	42.2	1418.9	42.8	0.842	570	1321.3	18.6	1279.9	52.8	1385.3	30.0	1316.0	34.2	1227.8	40.9	0.560
Health-conscious	113	1222.4	43.9	1744.7	138.7	1141.8	68.3	1125.2	70.7	1411.1	127.9	0.548	196	1139.0	31.7	1180.6	95.9	1084.2	51.7	1256.0	50.5	910.2	88.2	0.447
Denmark																								
Copenhagen	1356	1152.0	12.7			1162.6	19.8	1143.5	16.6	1136.0	107.9	0.158	1484	1009.3	11.6			1092.5	18.6	949.4	14.8	954.9	91.0	0.355
Aarhus	567	1220.8	19.6			1265.9	26.7	1176.7	28.6	1030.8	227.0	0.088	510	1109.5	19.7			1153.2	26.4	1058.7	29.8	929.4	152.6	0.057
Sweden																								
Malmö	1421	855.7	13.2			1007.6	34.3	886.8	19.4	752.8	18.0	0.019	1711	805.7	11.0			865.8	21.6	781.8	17.6	744.8	17.2	0.141
Umeå	1342	785.6	12.8	804.0	41.4	824.2	23.0	755.7	17.1	782.9	52.9	0.420	1567	704.0	11.2	792.5	26.1	733.9	19.4	643.7	16.9	694.7	50.8	0.212
Norway																								
South and East													1004	892.8	14.4	853.6	30.9	909.2	17.4	1000.1	38.4			0.088
North and West													793	894.5	16.0	914.7	33.6	912.9	19.2	906.6	48.8			0.195

^1^ Adjusted for total energy intake, weight, and height and weighted by season and day of recall. ^2^ SEM: standard error of the mean. If a group comprised fewer than 20 persons, mean intake is not presented.

**Table 4:** Fully adjusted mean ^1^ daily intake of coffee and tea (g/day) by education level and sex in the EPIC calibration study population based on 24-H Dietary Recall across EPIC centres ordered from south to north.

Country and Centre	Men						Women					
	*n*	All	None/Primary	Tech/Professional/Secondary	University	*p*-Trend	*n*	All	None/Primary	Tech/Professional/Secondary	University	*p*-Trend
Greece	1319	171.8 (13.2)	176.6 (19.1)	145.4 (27.8)	176.2 (23.2)	0.993	1361	170.6 (12.5)	162.2 (17.0)	158.6 (25.1)	181.7 (25.3)	0.425
Spain												
Granada	208	388.2 (32.1)	375.0 (42.5)	383.1 (79.8)	409.8 (60.5)	0.191	294	426.9 (26.0)	434.8 (27.7)	334.8 (93.1)	362.5 (118.2)	0.506
Murcia	243	300.3 (29.9)	266.0 (35.2)	385.3 (93.3)	374.9 (68.6)	0.384	304	390.6 (25.5)	384.0 (29.6)	360.5 (81.1)	430.6 (62.9)	0.547
Navarra	442	307.7 (22.2)	301.1 (26.1)	315.5 (47.7)	322.7 (82.1)	0.122	270	493.0 (27.0)	486.3 (29.5)	443.6 (89.7)	601.2 (97.3)	0.502
San Sebastian	488	269.8 (21.3)	251.8 (26.9)	281.3 (38.6)	339.1 (68.8)	0.118	242	464.7 (28.5)	478.6 (33.4)	454.3 (63.0)	361.6 (103.0)	0.207
Asturias	384	372.8 (23.8)	372.7 (29.6)	331.2 (49.7)	427.2 (63.4)	0.617	319	534.7 (24.9)	533.6 (27.7)	607.5 (72.6)	422.8 (87.1)	0.594
Italy												
Ragusa	167	221.2 (35.9)	200.2 (52.9)	241.0 (57.1)	221.6 (90.3)	0.648	137	201.6 (38.0)	224.3 (54.1)	173.8 (60.0)	192.0 (110.5)	0.566
Naples							403	297.5 (22.3)	287.9 (34.1)	305.2 (34.1)	283.0 (54.6)	0.865
Florence	269	269.4 (28.2)	264.6 (45.3)	278.1 (42.0)	256.0 (68.2)	0.747	780	328.2 (15.9)	314.1 (22.9)	330.6 (26.3)	357.6 (39.4)	0.088
Turin	676	260.2 (17.9)	242.8 (28.6)	270.0 (24.6)	270.6 (58.3)	0.322	392	312.4 (22.4)	299.7 (29.5)	323.4 (39.5)	338.4 (67.4)	0.082
Varese	327	392.0 (25.6)	422.2 (36.8)	370.3 (37.0)	279.4 (120.9)	0.100	794	404.1 (15.9)	408.9 (19.5)	384.4 (30.3)	407.6 (57.4)	0.969
France												
South coast							595	565.4 (18.2)	521.6 (49.0)	537.6 (25.1)	624.1 (30.6)	0.241
South							1358	649.5 (12.2)	549.0 (36.4)	626.7 (16.7)	711.7 (19.7)	0.016
Northeast							1984	658.6 (10.1)	574.0 (28.0)	652.0 (14.4)	694.4 (15.9)	0.108
Northwest							615	722.0 (17.9)	616.7 (48.3)	730.0 (23.6)	755.5 (32.8)	0.223
Germany												
Heidelberg	1031	897.6 (14.5)	854.8 (24.5)	855.8 (24.2)	995.5 (26.2)	0.330	1085	970.1 (13.6)	948.9 (26.1)	996.4 (19.0)	949.3 (28.1)	0.995
Potsdam	1233	844.0 (13.2)	829.3 (29.1)	811.5 (23.4)	871.3 (18.9)	0.521	1060	816.5 (13.7)	816.7 (26.8)	835.4 (19.4)	780.4 (27.3)	0.550
The Netherlands												
Bilthoven	1017	962.1 (15.0)	1031.4 (39.7)	928.9 (19.3)	1002.6 (27.9)	0.824	1071	951.3 (13.8)	894.5 (35.8)	930.9 (17.2)	1058.2 (28.8)	0.198
Utrecht							1869	1050.2 (10.4)	1030.6 (20.9)	1036.0 (13.2)	1138.0 (26.6)	0.305
United Kingdom												
General population	335	1470.9 (25.0)	1640.0 (63.0)	1500.2 (33.3)	1321.8 (47.0)	0.045	448	1312.3 (20.7)	1406.6 (43.8)	1319.9 (27.5)	1195.9 (44.2)	0.065
Health-conscious	84	1299.3 (49.6)		1119.2 (85.9)	1392.6 (60.2)		164	1186.3 (34.7)		1250.0 (53.2)	1143.9 (45.4)	
Denmark												
Copenhagen	1355	1153.2 (12.7)	1155.3 (23.2)	1135.5 (20.0)	1176.4 (22.5)	0.656	1484	1009.5 (11.6)	938.2 (22.3)	1020.9 (14.8)	1106.8 (32.3)	0.007
Aarhus	567	1221.6 (19.5)	1279.7 (34.3)	1176.8 (29.5)	1227.7 (38.5)	0.663	510	1109.9 (19.6)	1152.7 (36.0)	1079.0 (24.9)	1195.7 (65.8)	0.763
Sweden												
Malmö	1418	856.9 (13.1)	844.5 (18.9)	856.4 (22.0)	886.3 (25.9)	0.155	1708	805.8 (11.0)	780.7 (17.3)	809.5 (17.5)	836.4 (22.8)	0.012
Umeå	1338	787.5 (12.8)	785.2 (21.0)	761.3 (19.5)	847.8 (27.3)	0.506	1560	704.9 (11.2)	656.8 (21.2)	703.9 (16.7)	756.5 (21.4)	0.020
Norway												
South and East							1004	893.7 (14.3)	900.9 (34.3)	890.0 (17.3)	932.2 (34.8)	0.494
North and West							793	895.5 (16.0)	942.4 (33.8)	886.9 (19.5)	894.0 (44.6)	0.408

^1^ Adjusted for age, total energy intake, weight, and height and weighted by season and day of recall. If a group comprised fewer than 20 persons, mean intake is not presented.

**Table 5:** Total mean intake of energy and selected nutrients, amount of energy and selected nutrients from coffee and tea, and percentage contribution of coffee and tea to the total mean intake of energy and selected nutrients in the EPIC calibration study population based on 24-H Dietary Recall, by center ordered from south to north.

Country and Centre	Total Energy Intake (kcal) ^1^			Sugar (g) ^1^			Calcium (mg) ^1^			Magnesium (mg) ^1^			Phosphorus (mg) ^1^		
	Total Mean Intake (s.e ^3^)	From CT ^2^ (s.e ^3^)	%	Total Mean Intake (s.e ^3^)	From CT ^2^ (s.e ^3^)	%	Total Mean Intake (s.e ^3^)	From CT ^2^ (s.e ^3^)	%	Total Mean Intake (s.e ^3^)	From CT ^2^ (s.e ^3^)	%	Total Mean Intake (s.e ^3^)	From CT ^2^ (s.e ^3^)	%
Greece	1939.2 (14.0)	59.6 (1.7)	3.3	79.0 (1.0)	8.4 (0.3)	11.8	986.1 (9.0)	80.2 (2.7)	11.4	318.1 (2.3)	23.5 (0.9)	8.7	1789.4 (10.9)	68.1 (2.1)	5.3
Spain															
Granada	2142.0 (31.3)	153.1 (3.8)	7.9	102.6 (2.3)	19.9 (0.7)	21.5	1027.5 (20.3)	250.1 (6.1)	28.1	369.6 (5.2)	39.7 (1.9)	11.7	1402.8 (24.4)	191.0 (4.8)	15.6
Murcia	2328.4 (30.3)	133.3 (3.7)	6.5	117.0 (2.2)	18.7 (0.6)	18.4	1011.4 (19.6)	238.1 (5.9)	27.8	403.9 (5.1)	40.5 (1.9)	11.3	1456.4 (23.7)	183.0 (4.6)	18.9
Navarra	2294.0 (26.6)	140.0 (3.3)	6.7	96.2 (1.9)	18.4 (0.6)	20.9	908.3 (17.2)	254.2 (5.2)	33.0	360.3 (4.5)	41.9 (1.6)	13.0	1447.9 (20.7)	193.4 (4.1)	14.6
San Sebastian	2456.0 (26.3)	138.0 (3.2)	6.1	110.3 (1.9)	18.8 (0.6)	18.4	976.8 (17.0)	214.9 (5.1)	25.3	411.1 (4.4)	45.6 (1.6)	12.6	1707.1 (20.5)	179.2 (4.0)	11.6
Asturias	2292.6 (26.6)	170.3 (3.3)	8.2	114.1 (1.9)	22.3 (0.6)	21.6	1040.4 (17.2)	294.3 (5.2)	33.3	393.6 (4.5)	47.0 (1.6)	13.0	1659.2 (20.8)	224.8 (4.1)	15.1
Italy															
Ragusa	2284.5 (40.6)	81.1 (5.0)	3.7	93.8 (3.0)	15.1 (0.9)	16.2	752.0 (26.3)	76.5 (7.9)	12.2	370.3 (6.8)	30.0 (2.5)	9.2	1358.7 (31.7)	75.1 (6.2)	6.2
Naples	2214.5 (35.6)	99.1 (4.4)	5	91.6 (2.6)	17.7 (0.8)	22.0	852.0 (23.0)	137.1 (6.9)	23.8	316.1 (6.0)	43.0 (2.2)	16.2	1394.2 (27.7)	122.9 (5.4)	12.1
Florence	2183.1 (21.9)	88.9 (2.7)	4.4	89.6 (1.6)	12.8 (0.5)	15.6	798.1 (14.1)	134.2 (4.3)	23.8	328.2 (3.7)	38.5 (1.4)	13.4	1374.1 (17.0)	122.0 (3.3)	10.8
Turin	2202.3 (21.7)	99.5 (2.7)	4.7	103.0 (1.6)	17.7 (0.5)	17.4	866.6 (14.0)	95.7 (4.2)	15.4	335.0 (3.6)	34.7 (1.3)	11.6	1349.4 (16.9)	92.3 (3.3)	7.9
Varese	2274.7 (21.2)	138.6 (2.6)	6.6	104.3 (1.5)	22.3 (0.4)	23.2	877.6 (13.7)	152.2 (4.1)	24.3	322.7 (3.6)	44.0 (1.3)	16.0	1413.2 (16.5)	138.8 (3.2)	11.8
France															
South Coast	2316.0 (28.8)	78.7 (3.5)	3.7	99.9 (2.1)	10.3 (0.6)	10.9	1037.1 (18.6)	113.5 (5.6)	13.7	405.6 (4.8)	96.8 (1.8)	28.6	1500.2 (22.4)	92.7 (4.4)	7.4
South	2271.3 (19.4)	74.3 (2.4)	3.3	103.1 (1.4)	9.8 (0.4)	9.6	956.5 (12.5)	106.4 (3.8)	13.9	395.5 (3.2)	101.3 (1.2)	30.3	1450.6 (15.1)	88.2 (3.0)	7
Northeast	2338.5 (16.3)	70.3 (2.0)	3.1	104.9 (1.2)	9.0 (0.3)	8.6	969.8 (10.5)	97.8 (3.2)	12.0	414.2 (2.7)	112.9 (1.0)	32.7	1470.8 (12.7)	81.2 (2.5)	6.1
Northwest	2297.5 (28.5)	69.4 (3.5)	3.0	100.8 (2.1)	8.3 (0.6)	7.9	917.4 (18.5)	87.0 (5.6)	11.2	439.9 (4.8)	135.1 (1.8)	35.4	1461.5 (22.3)	76.1 (4.4)	5.6
Germany															
Heidelberg	2154.1 (15.5)	79.7 (1.9)	3.9	102.2 (1.1)	10.1 (0.3)	10.1	1005.8 (10.1)	104.9 (3.0)	13.0	430.1 (2.6)	48.9 (1.0)	12.9	1332.6 (12.1)	74.5 (2.4)	6.4
Potsdam	2186.7 (14.8)	54.4 (1.8)	2.7	116.0 (1.1)	6.1 (0.3)	5.8	858.2 (9.6)	47.2 (2.9)	7.2	392.9 (2.5)	40.0 (0.9)	11.4	1275.3 (11.6)	33.2 (2.3)	3.1
The Netherlands															
Bilthoven	2224.9 (15.8)	99.4 (1.9)	4.4	119.2 (1.2)	18.3 (0.3)	13.6	968.0 (10.3)	111.4 (3.1)	15.0	353.0 (2.7)	47.0 (1.0)	15.3	1562.0 (12.4)	71.0 (2.4)	5.2
Utrecht	2254.6 (17.4)	70.2 (2.1)	3.1	120.4 (1.3)	10.7 (0.4)	8.8	1124.2 (11.3)	123.8 (3.4)	13.9	363.4 (2.9)	44.2 (1.1)	13.4	1644.3 (13.6)	74.8 (2.7)	4.8
United Kingdom															
General population	2039.6 (22.7)	103.7 (2.8)	5.3	113.4 (1.7)	13.8 (0.5)	12.1	987.7 (14.7)	163.4 (4.4)	19.7	321.0 (3.8)	51.3 (1.4)	17.9	1407.5 (17.7)	157.2 (3.5)	12.5
Health-conscious	2070.1 (40.3)	68.8 (5.0)	3.4	117.3 (2.9)	7.2 (0.9)	6.3	887.0 (26.1)	104.0 (7.9)	14.7	396.3 (6.8)	39.1 (2.5)	11.2	1314.8 (31.4)	60.5 (6.2)	5.3
Denmark															
Copenhagen	2235.4 (13.7)	49.3 (1.7)	2.3	99.5 (1.0)	6.0 (0.3)	5.0	960.0 (8.8)	43.7 (2.7)	6.1	365.1 (2.3)	49.9 (0.8)	15.3	1555.3 (10.6)	38.2 (2.1)	2.8
Aarhus	2383.2 (21.7)	42.9 (2.7)	2.0	105.5 (1.6)	4.0 (0.5)	3.6	1050.4 (14.0)	38.3 (4.2)	4.8	384.1 (3.6)	56.7 (1.3)	16.0	1632.9 (16.9)	34.5 (3.3)	2.4
Sweden															
Malmö	2039.6 (13.2)	50.2 (1.6)	2.6	96.0 (1.0)	6.2 (0.3)	6.4	869.4 (8.5)	45.4 (2.6)	7.4	304.8 (2.2)	39.3 (0.8)	14.7	1300.2 (10.3)	33.2 (2.0)	2.9
Umeå	2131.0 (13.3)	41.0 (1.6)	2.0	102.3 (1.0)	5.6 (0.3)	5.5	989.9 (8.6)	39.8 (2.6)	5.3	323.8 (2.2)	36.2 (0.8)	12.5	1417.0 (10.4)	30.0 (2.0)	2.4
Norway															
South and East	2092.8 (23.2)	29.2 (2.9)	1.2	99.3 (1.7)	2.9 (0.5)	2.7	814.7 (15.0)	33.9 (4.5)	5.5	363.3 (3.9)	40.6 (1.4)	12.7	1482.3 (18.1)	26.4 (3.5)	1.6
North and West	2075.5 (25.8)	29.0 (3.2)	1.3	100.4 (1.9)	2.8 (0.5)	2.5	815.2 (16.7)	29.4 (5.8)	3.3	364.0 (4.3)	41.3 (1.8)	9.4	1487.4 (20.1)	22.0 (4.5)	1.6

^1^ Adjusted for sex, age, height, and weight and weighted by season and day of recall. ^2^ Coffee and tea. ^3^ s.e.: standard error.

## Supplementary Materials

The following are available online at http://www.mdpi.com/2072-6643/10/6/725/s1. Figure S1: Percentages of consumers and nonconsumers of coffee and tea among men the day of the 24-h recall by centre; Figure S2: Percentages of consumers and nonconsumers of coffee and tea among women the day of the 24-h recall by centre; Figure S3: Percentage of consumers of coffee with caffeine and decaffeinated coffee among men who consumed coffee the day of the 24-h recall by centre; Figure S4: Percentage of consumers of coffee with caffeine and decaffeinated coffee among women who consumed coffee the day of the 24-h recall by centre; Table S1: Mean daily intake of coffee and tea (g/day) by type and country in the EPIC calibration study population based on 24-HDR among men and women; Table S2: Fully adjusted mean daily intake of coffee and tea (g/day) by smoking status and sex in the EPIC calibration study population based on 24-HDR across EPIC centres ordered from south to north; Table S3: Fully adjusted mean daily intake of coffee and tea (g/day) by physical activity level and sex in the EPIC calibration study population based on 24-HDR across EPIC centres ordered from south to north; Table S4: Fully adjusted mean daily intake of coffee and tea (g/day) by BMI group and sex in the EPIC calibration study population based on 24-HDR across EPIC centres ordered from south to north.
