# Supplementary material for: Coffee and Tea Consumption and the Contribution of Their Added Ingredients to Total Energy and Nutrient Intakes in 10 European Countries: Benchmark Data from the Late 1990s
Source: Nutrients. 2018 Jun 5;10(6):725. doi: 10.3390/nu10060725 (PMC6024313; doi:10.3390/nu10060725)
Supplement: Supplementary file 1 [file nutrients-10-00725-s001.pdf]

Supplementary material – *Nutrients*

Coffee and Tea Consumption and the Contribution of Their Added Ingredients to Total Energy and Nutrient Intakes in 10 European Countries: Nineteen Nineties' Benchmark Data.

Edwige Landais [1], Aurélie Moskal [2], Amy Mullee [2,3] Geneviève Nicolas [2], Marc Gunter [2], Inge Huybrechts [2], Kim Overvad [4], Nina Roswall [5], Aurélie Affret [6], Guy Fagherazzi [6], Yahya Mahamat-Saleh [6], Verena Katzke [7], Tilman Kühn [7], Carlo La Vecchia [8,9], Antonia Trichopoulou [8], Elissavet Valanou [8], Calogero Saieva [10], Maria Santucci de Magistris [11], Sabina Sieri [12], Tonje Braaten [13], Guri Skeie [13], Elisabete Weiderpass [14,15,16,17], Eva Ardanaz [18,19], Maria-Dolores Chirlaque [19,20,21], Jose Ramon Garcia [22], Paula Jakszyn [23], Miguel Rodríguez-Barranco [19,24,25], Louise Brunkwall [26], Ena Huseinovic [27], Lena Nilsson [28], Peter Wallström [26], Bas Bueno-de-Mesquita [29,30], Petra H. Peeters [31], Dagfinn Aune [29], Tim Key [32], Marleen Lentjes [33], Elio Riboli [29], Nadia Slimani [2], Heinz Freisling [2]*

Contact:

Nutrition and Metabolism Section, International Agency for Research on Cancer, Lyon, France; MoskalA@iarc.fr (A.M.); nicolasg@iarc.fr (G.N.); gunterM@iarc.fr (M.G.); huybrechtsi@iarc.fr (I.H.); n.popovic@orange.fr (N.S.); freislingh@iarc.fr (H.F.)

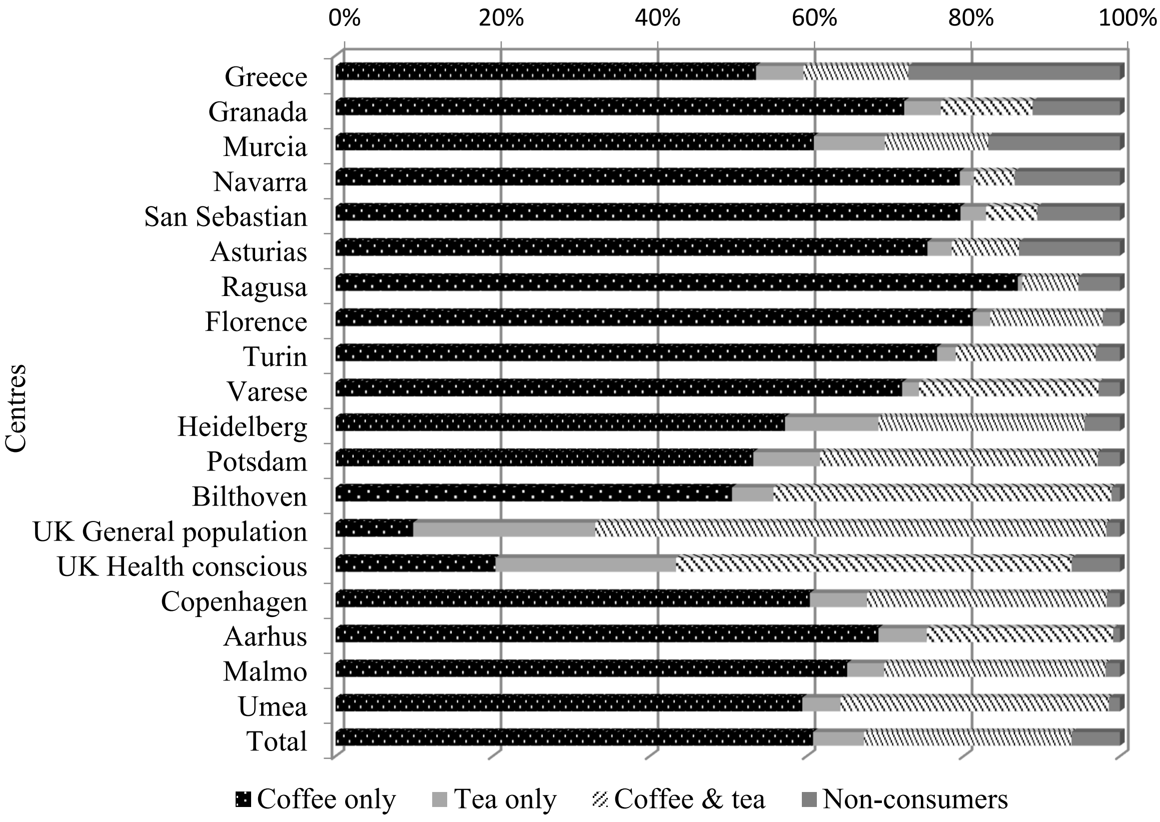

Figure S1. Percentages of consumers and non-consumers of coffee and tea among men the day of the 24-h recall by centre.

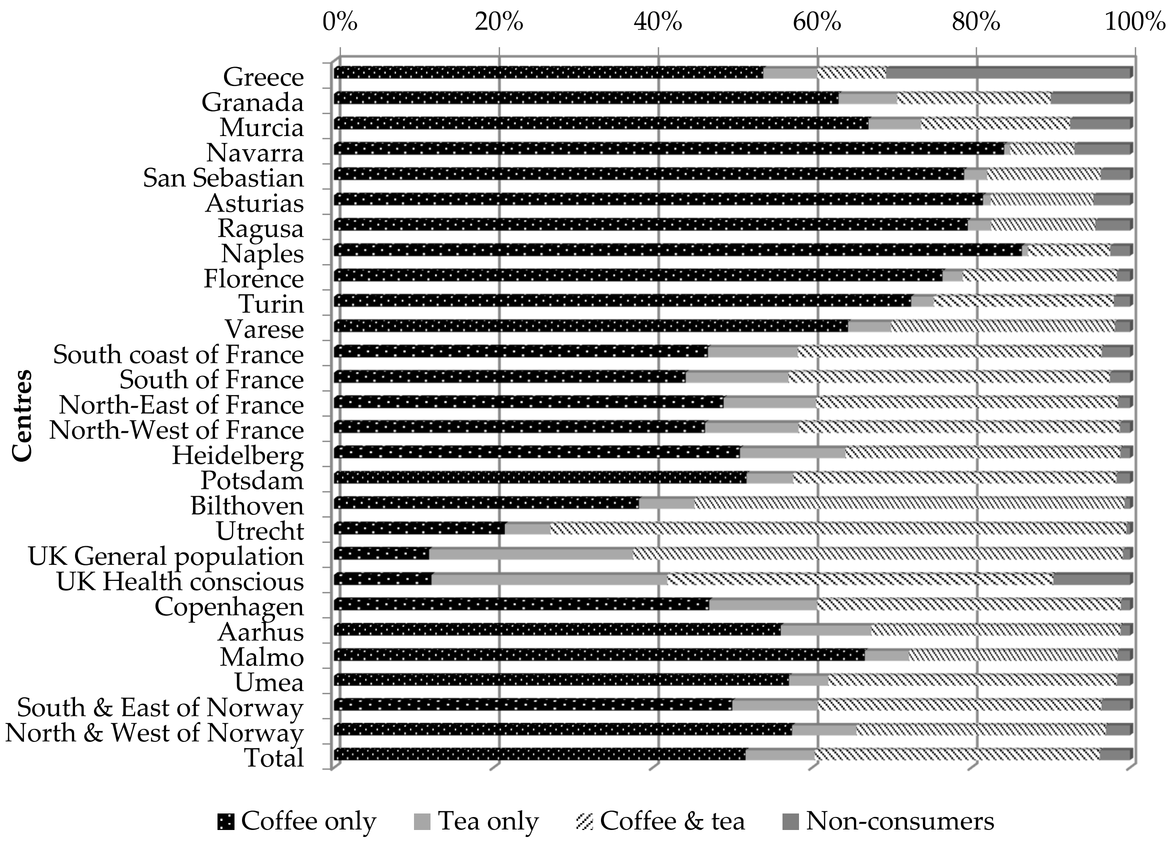

Figure S2. Percentages of consumers and non-consumers of coffee and tea among women the day of the 24-h recall by centre.

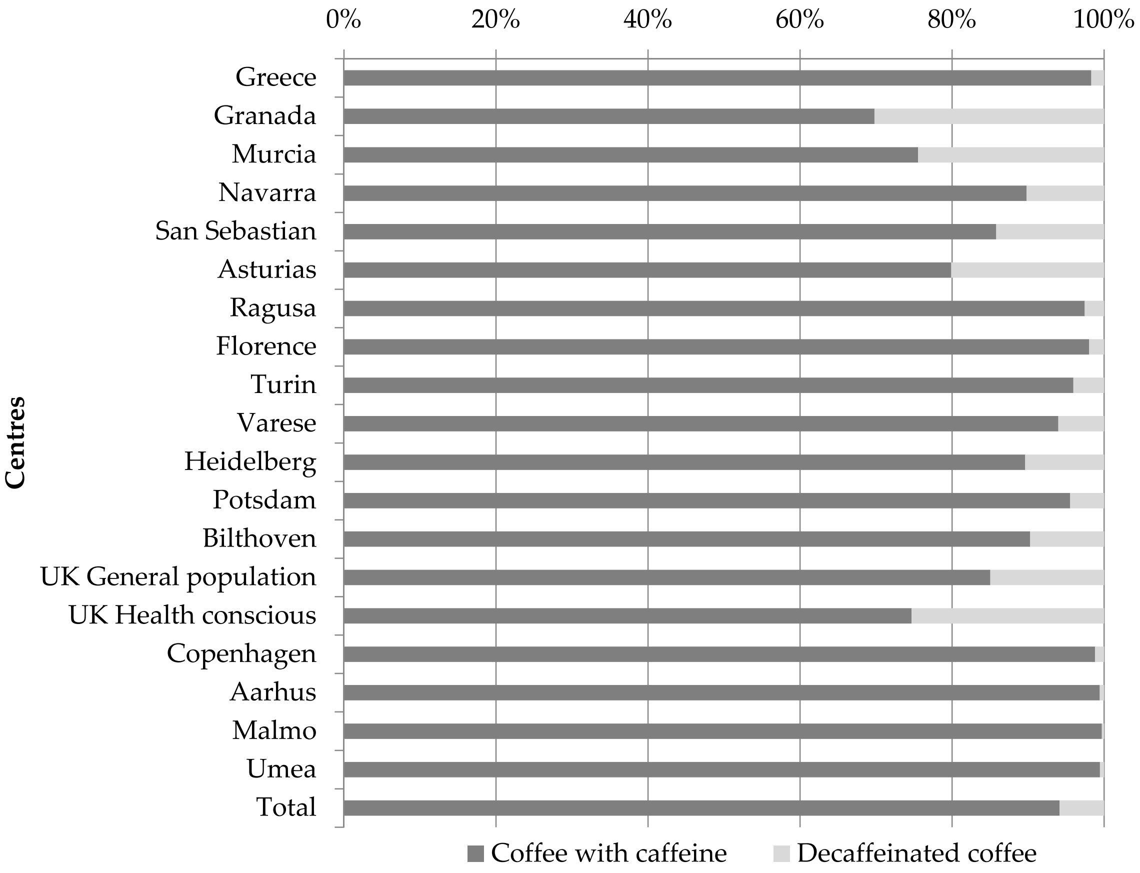

Figure S3. Percentage of consumers of coffee with caffeine and decaffeinated coffee among men who consumed coffee the day of the 24-h recall by centre.

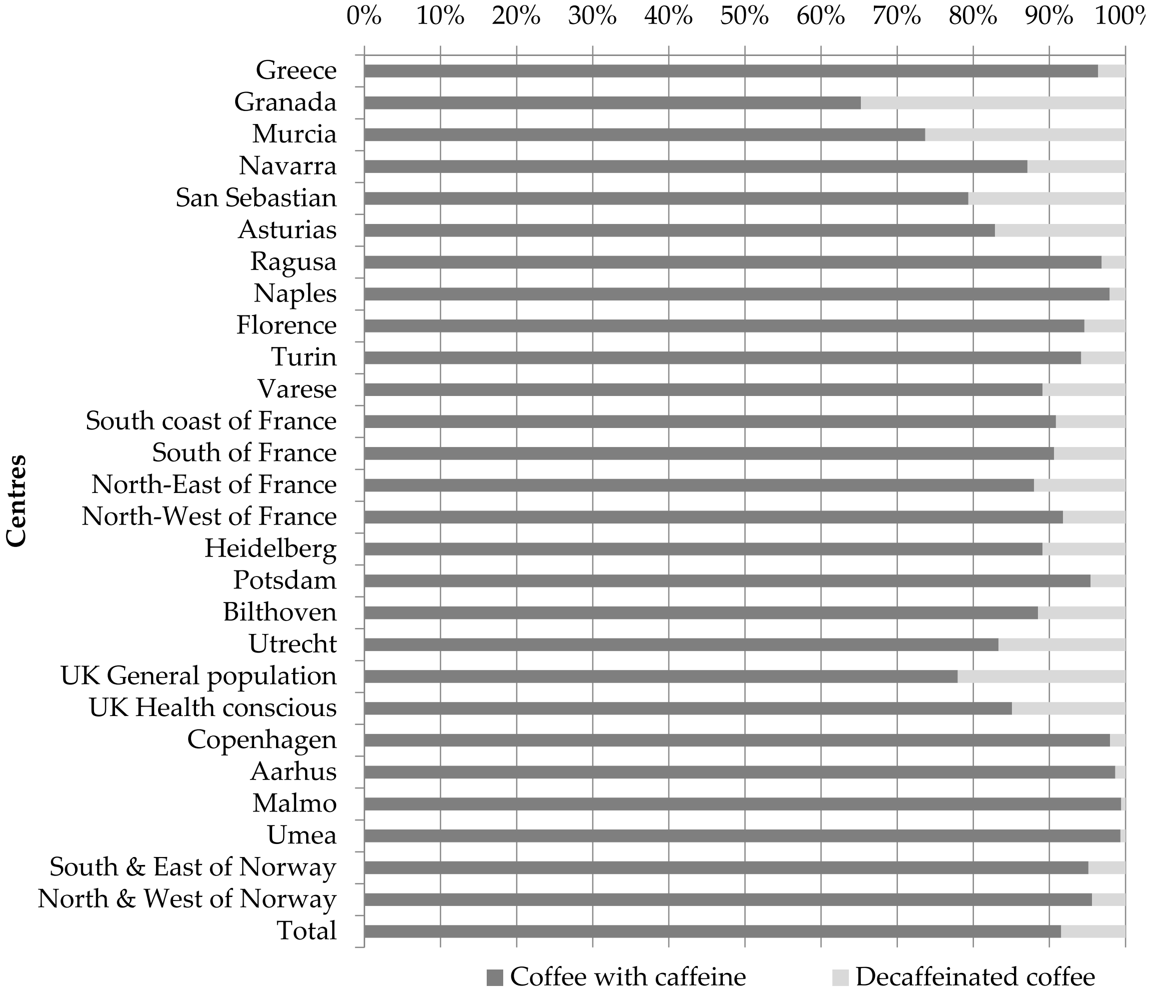

Figure S4. Percentage of consumers of coffee with caffeine and decaffeinated coffee among women who consumed coffee the day of the 24-h recall by centre.

Table S1. Mean daily intake of coffee and tea (g/day) by type and country in the EPIC calibration study population based on 24-HDR among men and women

			Coffee & Tea*		Coffee		Tea*		Herbal tea		Chicory & substitutes	
Country	Sex	n	fully adjusted mean[1]	SEM[2]	fully adjusted mean[1]	SEM[2]	fully adjusted mean[1]	SEM[2]	fully adjusted mean[1]	SEM[2]	fully adjusted mean[1]	SEM[2]
Italy	Male	1442	284.2	12.4	193.1	11.5	56.6	8.6	12.1	4.2	22.3	1.7
Spain	Male	1777	325.9	11.5	252.6	10.6	21.5	7.9	29.0	3.9	22.9	1.6
United Kingdom	Male	518	1413.4	20.5	510.6	18.9	849.0	14.1	37.2	6.9	16.5	2.8
The Netherlands	Male	1020	966.5	15.1	704.0	13.9	235.9	10.4	21.2	5.1	5.4	2.1
Greece	Male	1324	171.0	13.2	104.1	12.2	47.3	9.1	18.4	4.4	1.2	1.8
Germany	Male	2267	866.5	9.8	586.7	9.1	142.4	6.8	127.4	3.3	10.1	1.3
Sweden	Male	2763	811.3	9.4	666.3	8.7	147.7	6.5				
Denmark	Male	1923	1174.3	10.7	936.5	9.9	213.7	7.4	24.1	3.6		
France	Female	4735	650.2	6.7	310.1	5.6	209.4	5.3	61.2	3.0	69.5	1.8
Italy	Female	2510	343.5	9.1	229.4	7.5	70.3	7.2	20.2	4.1	23.6	2.4
Spain	Female	1443	471.7	12.0	373.2	9.9	22.2	9.4	51.7	5.4	24.6	3.1
United Kingdom	Female	766	1282.5	16.1	458.5	13.3	745.3	12.6	55.3	7.2	23.4	4.2
The Netherlands	Female	2946	1012.4	8.3	578.3	6.9	384.2	6.5	40.4	3.7	9.6	2.2
Greece	Female	1368	172.3	12.6	93.8	10.4	54.8	9.9	21.0	5.6	2.7	3.3
Germany	Female	2147	902.3	9.8	540.3	8.1	152.7	7.7	196.0	4.4	13.3	2.6
Sweden	Female	3278	752.1	8.0	586.8	6.6	155.2	6.2	10.2	3.6		
Denmark	Female	1994	1029.5	10.1	669.3	8.4	290.4	7.9	69.0	4.5	0.8	2.6
Norway	Female	1797	900.1	11.0	667.3	9.1	169.4	8.6	62.2	4.9	1.2	2.9

* Either green or black tea, herbal tea excluded.

[1] Adjusted for age, total energy intake, weight, height and weighted by season and day of recall.

[2] SEM standard error of the mean.

Table S2. Fully adjusted mean[1] daily intake of coffee and tea (g/day) by smoking status and sex in the EPIC calibration study population based on 24-HDR across EPIC centres ordered from South to North

Country & Centre	Men						Women					
	n	All	Never smoker	Former smoker	Current smoker	p-trend	n	All	Never smoker	Former smoker	Current smoker	p-trend
Greece	1243	175.2 (13.7)	159.0 (24.2)	208.8 (21.6)	147.9 (23.0)	0.891	1306	167.8 (12.8)	156.0 (14.5)	186.6 (48.5)	198.3 (29.0)	0.160
Spain												
Granada	214	387.6 (32.0)	342.8 (54.0)	406.7 (48.8)	416.2 (63.7)	0.257	300	425.7 (25.8)	405.4 (27.7)	644.2 (112.9)	481.9 (81.5)	0.797
Murcia	243	302.1 (30.1)	292.9 (54.0)	294.9 (54.5)	317.2 (46.4)	0.287	303	390.0 (25.7)	381.8 (29.0)	420.7 (93.9)	419.4 (63.7)	0.352
Navarra	444	309.3 (22.4)	277.7 (37.0)	331.6 (50.7)	325.9 (32.3)	0.395	271	491.0 (27.0)	468.0 (31.8)	591.3 (79.4)	518.6 (63.6)	0.732
San Sebastian	490	270.0 (21.5)	221.2 (36.7)	302.0 (41.1)	294.2 (32.4)	0.389	244	468.0 (28.5)	494.1 (33.2)	381.5 (74.8)	431.3 (76.0)	0.624
Asturias	385	380.8 (23.9)	376.8 (40.0)	363.9 (40.6)	404.9 (41.5)	0.532	324	532.6 (24.8)	514.7 (28.2)	691.2 (92.9)	549.6 (59.0)	0.881
Italy												
Ragusa	167	221.7 (36.2)	223.1 (65.2)	224.5 (51.6)	223.2 (74.9)	0.946	137	200.7 (38.1)	200.0 (57.9)	235.2 (84.2)	189.7 (61.4)	0.862
Naples							403	297.1 (22.3)	296.1 (31.3)	281.5 (49.1)	306.1 (39.9)	0.736
Florence	266	269.3 (28.6)	275.7 (54.1)	264.9 (40.4)	276.2 (56.9)	0.975	783	327.9 (15.9)	318.2 (22.7)	361.3 (32.1)	315.7 (29.8)	0.970
Turin	661	261.1 (18.2)	259.3 (34.0)	259.0 (26.7)	271.5 (34.5)	0.344	392	312.1 (22.5)	306.3 (28.5)	300.8 (47.2)	352.3 (54.3)	0.395
Varese	325	391.7 (25.9)	381.6 (42.7)	369.7 (40.1)	448.4 (52.5)	0.423	795	403.9 (15.9)	409.2 (18.7)	407.0 (42.1)	377.3 (39.5)	0.294
France												
South Coast							598	570.7 (18.3)	543.6 (20.7)	632.6 (43.7)	727.6 (71.3)	0.012
South							1367	650.8 (12.2)	629.2 (14.2)	699.0 (25.7)	731.6 (45.5)	0.132
North East							1955	656.3 (10.2)	631.5 (11.7)	720.0 (22.9)	745.3 (38.0)	0.198
North West							607	724.4 (18.1)	707.6 (21.5)	756.4 (37.7)	788.1 (63.9)	0.078
Germany												
Heidelberg	1034	897.2 (14.6)	816.9 (24.9)	907.3 (21.6)	998.6 (30.2)	0.002	1087	968.4 (13.7)	962.6 (18.8)	925.2 (25.1)	1058.3 (29.7)	0.509
Potsdam	1233	844.1 (13.3)	830.4 (23.4)	827.9 (19.2)	898.8 (28.0)	0.354	1058	816.5 (13.7)	812.4 (17.0)	767.7 (29.1)	917.4 (35.8)	0.521
The Netherlands												
Bilthoven	1015	960.5 (15.2)	936.0 (29.7)	929.2 (23.2)	1015.5 (24.4)	0.379	1071	949.5 (13.9)	885.9 (23.1)	950.2 (23.9)	1022.4 (23.4)	0.021
Utrecht							1869	1050.2 (10.5)	1018.7 (15.1)	1063.2 (17.5)	1090.8 (23.2)	0.085
United Kingdom												
General Population	399	1468.6 (23.4)	1313.2 (38.0)	1456.1 (34.0)	1827.7 (55.2)	0.160	562	1320.8 (18.7)	1274.3 (23.8)	1263.0 (34.5)	1774.2 (59.1)	0.346
Health Conscious	113	1222.5 (44.1)	1069.9 (68.8)	1214.6 (67.4)	1569.6 (99.5)	0.152	195	1140.9 (31.8)	1173.4 (38.9)	1069.4 (55.8)	1275.9 (206.8)	0.669
Denmark												
Copenhagen	1355	1152.7 (12.8)	1043.2 (22.9)	1109.5 (20.3)	1305.8 (22.2)	0.177	1484	1009.3 (11.6)	951.0 (16.3)	1016.9 (22.1)	1117.3 (23.5)	0.076
Aarhus	564	1221.2 (19.7)	1038.4 (37.6)	1149.4 (31.4)	1435.7 (32.5)	0.159	509	1109.5 (19.7)	1059.3 (28.6)	1113.1 (37.2)	1194.4 (38.3)	0.075
Sweden												
Malmö	1421	856.5 (13.2)	775.8 (22.3)	846.5 (19.2)	964.6 (24.7)	0.092	1711	805.7 (11.0)	770.7 (15.6)	773.9 (20.2)	901.0 (22.0)	0.320

Umeå	1320	787.7 (13.0)	735.8 (17.6)	820.0 (23.0)	886.3 (30.6)	0.044	1551	705.2 (11.3)	672.8 (13.9)	716.6 (27.2)	811.5 (26.6)	0.134
Norway												
South & East							952	892.4 (14.7)	799.6 (23.5)	871.7 (24.3)	1060.3 (27.4)	0.161
North & West							752	903.0 (16.4)	844.2 (27.1)	819.9 (26.8)	1095.2 (30.1)	0.384

[1]Adjusted for age, total energy intake, weight, height and weighted by season and day of recall.
If a group comprised fewer than 20 persons, mean intake is not presented.

Table S3. Fully adjusted mean[1] daily intake of coffee and tea (g/day) by physical activity level and sex in the EPIC calibration study population based on 24-HDR across EPIC centres ordered from South to North

Country & Centre	Men							Women						
	n	All	Inactive	Moderately Inactive	Moderately Active	Active	p-trend	n	All	Inactive	Moderately Inactive	Moderately Active	Active	p-trend
Greece	1323	170.7 (13.4)	154.4 (33.4)	170.8 (21.3)	178.4 (20.8)	144.8 (47.6)	0.823	1368	164.9 (12.3)	187.8 (47.0)	160.9 (27.3)	167.8 (15.0)	133.6 (37.5)	0.103
Spain														
Granada	214	387.0 (32.2)	430.3 (70.6)	327.1 (58.0)	406.4 (52.3)	395.5 (91.9)	0.926	300	422.1 (25.4)	357.7 (171.0)	427.6 (91.5)	425.1 (27.1)	373.8 (134.6)	0.834
Murcia	243	302.1 (30.3)	342.5 (58.8)	315.0 (54.0)	271.6 (55.3)	258.1 (81.0)	0.017	304	389.0 (25.2)	559.5 (104.1)	421.5 (63.0)	369.2 (29.4)	390.2 (102.7)	0.156
Navarra	444	309.4 (22.5)	323.5 (51.3)	337.5 (41.4)	287.0 (36.5)	289.2 (56.7)	0.212	271	488.6 (26.6)	510.5 (112.4)	443.2 (76.9)	496.1 (30.0)	456.1 (122.2)	0.555
San Sebastian	490	272.2 (21.6)	300.9 (44.6)	269.2 (43.7)	280.6 (34.3)	219.2 (53.5)	0.132	244	466.6 (28.0)	497.1 (101.5)	497.5 (64.0)	443.2 (35.3)	531.7 (83.7)	0.825
Asturias	386	380.0 (24.0)	379.8 (57.0)	389.6 (42.0)	362.9 (42.4)	389.9 (54.6)	0.961	324	530.3 (24.3)	503.6 (106.3)	584.4 (67.7)	521.1 (28.2)	548.8 (83.0)	0.735
Italy														
Ragusa	168	222.5 (36.4)	216.4 (67.2)	256.1 (65.2)	210.6 (64.2)	170.7 (118.9)	0.326	137	199.5 (37.5)	207.1 (130.0)	243.6 (86.0)	198.2 (46.7)	122.6 (120.8)	0.241
Naples								403	294.7 (21.9)	293.0 (59.2)	291.6 (35.1)	288.1 (33.7)	355.5 (84.4)	0.266
Florence	271	269.6 (28.5)	270.5 (54.8)	263.0 (48.8)	293.1 (51.3)	214.9 (95.3)	0.464	783	324.0 (15.6)	307.3 (48.6)	336.2 (31.8)	321.6 (20.4)	325.0 (55.6)	0.581
Turin	676	260.0 (18.1)	264.7 (35.6)	257.5 (30.8)	270.5 (32.7)	226.1 (56.7)	0.329	392	308.5 (22.1)	319.6 (64.7)	353.9 (49.8)	309.9 (29.2)	220.5 (62.6)	0.225
Varese	327	391.8 (26.0)	335.8 (67.0)	394.2 (42.1)	413.1 (41.8)	374.5 (86.3)	0.473	795	400.6 (15.6)	417.4 (50.5)	380.6 (36.2)	402.3 (19.5)	409.5 (51.2)	0.984
France														
South Coast								620	563.6 (17.6)	555.2 (45.8)	560.1 (21.9)	602.0 (40.3)	393.3 (110.8)	0.377
South								1425	647.9 (11.7)	629.5 (29.5)	651.5 (14.4)	648.3 (27.7)	652.4 (81.4)	0.214
North East								2059	653.1 (9.7)	677.3 (22.8)	646.3 (12.1)	647.4 (23.6)	705.1 (79.4)	0.610
North West								631	719.5 (17.5)	755.5 (45.7)	726.7 (21.1)	657.0 (42.8)	707.5 (137.9)	0.335
Germany														
Heidelberg	1034	896.8 (14.7)	967.4 (32.8)	852.1 (26.3)	899.9 (23.2)	892.3 (45.7)	0.521	1087	965.8 (13.5)	942.9 (34.1)	1010.2 (23.4)	938.3 (20.1)	995.8 (44.0)	0.693
Potsdam	1233	843.7 (13.4)	942.4 (35.4)	786.6 (28.1)	856.7 (18.2)	765.5 (42.1)	0.256	1060	812.5 (13.5)	803.1 (29.7)	792.0 (24.3)	830.1 (20.0)	840.5 (65.4)	0.144
The Netherlands														
Bilthoven	1013	961.3 (15.3)	991.6 (42.7)	977.8 (31.5)	953.7 (21.8)	952.0 (33.7)	0.042	1071	946.5 (13.7)	920.8 (48.2)	1074.1 (29.6)	918.8 (17.9)	903.1 (34.5)	0.665
Utrecht								1869	1046.5 (10.3)	1148.6 (42.5)	1077.3 (20.8)	1017.1 (14.1)	1056.0 (23.3)	0.210
United Kingdom														
General Population	402	1457.0 (23.4)	1306.5 (58.9)	1457.4 (43.8)	1476.6 (36.9)	1555.5 (58.3)	0.049	568	1313.7 (18.3)	1313.2 (45.5)	1369.4 (33.3)	1252.0 (27.9)	1404.1 (55.3)	0.700
Health Conscious	112	1215.3 (44.5)	1469.7 (112.7)	1107.0 (84.0)	1108.5 (67.9)	1468.0 (114.7)	0.998	196	1134.9 (31.2)	1172.5 (64.1)	1074.1 (60.9)	1121.6 (45.8)	1438.2 47.7)	0.330
Denmark														
Copenhagen	1356	1151.6 (12.9)	1194.3 (24.5)	1157.9 (24.5)	1146.8 (23.1)	1078.6 (32.1)	0.043	1484	1005.5 (11.4)	1057.4 (21.2)	980.2 (19.0)	998.8 (20.9)	948.3 (44.5)	0.131
Aarhus	567	1221.1 (19.8)	1169.9 (39.5)	1147.6 (36.5)	1286.9 (33.8)	1317.1 (54.9)	0.109	510	1107.9 (19.4)	1103.9 (39.5)	1040.7 (31.6)	1179.0 (33.8)	1157.0 (75.6)	0.376

Sweden													
Malmö	1421	852.2 (13.4)	926.7 (29.0)	838.6 (19.7)	813.1 (21.9)	971.0 (63.4)	0.813	1711	800.0 (10.9)	817.9 (25.0)	819.8 (16.9)	767.8 (17.4)	805.3 (49.7)	0.519

[1]Adjusted for age, total energy intake, weight, height and weighted by season and day of recall.
If a group comprised fewer than 20 persons, mean intake is not presented.
No physical activity level was measured in Norway, and Umeå.

Table S4. Fully adjusted mean[1] daily intake of coffee and tea (g/day) by BMI groups and sex in the EPIC calibration study population based on 24-HDR across EPIC centres ordered from South to North

Country & Centre	Men						Women					
	n	All	BMI < 25kg/m²	BMI 25-30kg/m²	BMI ≥30kg/m²	p-trend	n	All	BMI < 25kg/m²	BMI 25-30kg/m²	BMI ≥30kg/m²	p-trend
Greece	1324	154.4 (12.9)	136.4 (30.3)	150.2 (17.3)	172.3 (24.4)	0.084	1368	144.7 (12.1)	143.3 (24.8)	141.6 (19.3)	148.1 (19.6)	0.500
Spain												
Granada	214	365.0 (31.8)	275.4 (121.6)	387.8 (38.5)	327.7 (62.4)	0.692	300	402.0 (25.6)	437.4 (50.6)	395.5 (37.3)	380.1 (48.7)	0.167
Murcia	243	277.8 (29.8)	255.0 (62.6)	264.8 (40.0)	333.9 (62.9)	0.261	304	369.2 (25.5)	371.0 (44.9)	394.3 (40.4)	333.4 (47.6)	0.581
Navarra	444	287.0 (22.1)	296.0 (55.0)	291.1 (28.6)	272.0 (44.2)	0.208	271	473.3 (27.0)	497.4 (44.0)	481.0 (41.0)	410.6 (60.9)	0.220
San Sebastian	490	254.7 (21.3)	267.6 (44.6)	250.8 (26.9)	258.2 (51.6)	0.625	244	457.1 (28.4)	437.1 (39.9)	467.1 (47.0)	508.4 (78.3)	0.058
Asturias	386	360.0 (23.7)	368.1 (56.2)	367.7 (30.6)	334.9 (48.9)	0.328	324	515.0 (24.7)	545.0 (42.7)	489.3 (34.8)	533.0 (59.9)	0.869
Italy												
Ragusa	168	200.1 (35.9)	175.1 (81.1)	212.3 (45.0)	187.9 (85.6)	0.780	137	172.7 (38.0)	123.1 (52.6)	231.0 (64.7)	217.1 (101.3)	0.409
Naples							403	272.3 (22.1)	260.0 (39.4)	275.6 (35.0)	281.4 (41.0)	0.165
Florence	271	265.1 (28.2)	275.2 (46.5)	250.9 (39.8)	294.6 (77.2)	0.708	783	319.0 (15.9)	340.8 (22.3)	302.3 (26.3)	282.0 (43.2)	0.113
Turin	676	251.2 (17.9)	260.2 (31.4)	250.3 (23.9)	234.7 (50.7)	0.083	392	300.6 (22.4)	326.5 (30.8)	277.1 (39.4)	260.1 (57.9)	0.175
Varese	327	384.4 (25.7)	435.4 (41.8)	356.3 (34.7)	331.8 (90.9)	0.188	795	387.7 (15.7)	389.8 (22.0)	379.9 (26.7)	398.8 (41.3)	0.684
France												
South coast							620	568.1 (17.9)	582.4 (20.0)	552.0 (45.2)	407.9 (75.4)	0.229
South							1425	653.0 (11.8)	656.2 (13.5)	663.2 (25.7)	527.9 (61.2)	0.363
North-East							2059	657.8 (9.8)	654.0 (11.7)	669.0 (20.0)	656.1 (41.6)	0.920
North-West							631	722.6 (17.7)	731.5 (20.2)	696.1 (39.3)	680.1 (93.0)	0.137
Germany												
Heidelberg	1034	901.0 (14.6)	1028.2 (25.4)	853.8 (20.5)	805.2 (34.2)	0.200	1087	972.8 (13.6)	986.7 (17.7)	967.8 (25.8)	926.0 (36.2)	0.136
Potsdam	1233	843.6 (13.3)	899.5 (25.1)	838.9 (18.2)	779.6 (29.6)	0.004	1060	811.6 (13.7)	796.1 (20.2)	862.1 (23.1)	758.6 (31.1)	0.767
The Netherlands												
Bilthoven	1020	967.8 (15.1)	997.7 (25.4)	960.5 (20.4)	933.7 (38.4)	0.060	1076	953.9 (13.8)	949.7 (19.2)	962.4 (23.1)	951.6 (35.0)	0.913
Utrecht							1870	1059.5 (10.4)	1071.0 (15.1)	1077.0 (16.9)	986.4 (25.3)	0.371
United Kingdom												

General population	405	1475.2 (23.1)	1540.7 (39.3)	1452.3 (31.5)	1391.0 (65.4)	0.066	570	1320.6 (18.6)	1249.5 (25.9)	1435.8 (31.3)	1290.7 (50.5)	0.865
Health conscious	113	1246.2 (43.7)	1192.1 (49.8)	1487.8 (94.6)	803.1 (300.5)	0.617	196	1146.9 (31.7)	1124.0 (35.7)	1301.4 (76.9)	968.9 (148.7)	0.692
Denmark												
Copenhagen	1356	1163.4 (12.6)	1184.1 (20.9)	1150.3 (18.1)	1155.9 (32.3)	0.432	1484	1018.5 (11.5)	1039.6 (16.1)	1022.3 (19.6)	935.3 (30.1)	0.235
Aarhus	567	1229.0 (19.6)	1206.6 (33.5)	1252.2 (26.8)	1196.9 (52.7)	0.895	510	1116.3 (19.7)	1129.3 (25.5)	1109.9 (36.4)	1067.9 (57.3)	0.133
Sweden												
Malmö	1421	870.9 (13.0)	838.9 (21.4)	879.6 (17.4)	907.4 (33.6)	0.069	1711	813.6 (11.0)	821.5 (15.7)	811.4 (17.9)	792.4 (26.9)	0.110
Umeå	1342	798.8 (12.7)	807.3 (20.9)	785.4 (17.6)	830.4 (37.3)	0.656	1567	709.3 (11.2)	697.0 (16.1)	733.4 (18.3)	688.0 (29.7)	0.880
Norway												
South & East							1004	910.7 (14.2)	918.2 (16.9)	890.1 (28.6)	912.5 (59.1)	0.877
North & West							793	907.9 (16.0)	925.8 (19.9)	888.6 (29.5)	834.7 (57.2)	0.067

[1]Adjusted for age, total energy intake, weight, height and weighted by season and day of recall.

If a group comprised fewer than 20 persons, mean intake is not presented.
